# Applications and Concerns of ChatGPT and Other Conversational Large Language Models in Health Care: Systematic Review

**DOI:** 10.2196/22769

**Published:** 2024-11-07

**Authors:** Leyao Wang, Zhiyu Wan, Congning Ni, Qingyuan Song, Yang Li, Ellen Clayton, Bradley Malin, Zhijun Yin

**Affiliations:** 1 Department of Computer Science Vanderbilt University Nashville, TN United States; 2 Department of Biomedical Informatics Vanderbilt University Medical Center Nashville, TN United States; 3 School of Biomedical Engineering ShanghaiTech University Shanghai China; 4 Department of Pediatrics Vanderbilt University Medical Center Nashville, TN United States; 5 School of Law Vanderbilt University Medical Center Nashville, TN United States; 6 Department of Biostatistics Vanderbilt University Medical Center Nashville, TN United States

**Keywords:** large language model, ChatGPT, artificial intelligence, natural language processing, health care, summarization, medical knowledge inquiry, reliability, bias, privacy

## Abstract

**Background:**

The launch of ChatGPT (OpenAI) in November 2022 attracted public attention and academic interest to large language models (LLMs), facilitating the emergence of many other innovative LLMs. These LLMs have been applied in various fields, including health care. Numerous studies have since been conducted regarding how to use state-of-the-art LLMs in health-related scenarios.

**Objective:**

This review aims to summarize applications of and concerns regarding conversational LLMs in health care and provide an agenda for future research in this field.

**Methods:**

We used PubMed, ACM, and the IEEE digital libraries as primary sources for this review. We followed the guidance of PRISMA (Preferred Reporting Items for Systematic Reviews and Meta-Analyses) to screen and select peer-reviewed research articles that (1) were related to health care applications and conversational LLMs and (2) were published before September 1, 2023, the date when we started paper collection. We investigated these papers and classified them according to their applications and concerns.

**Results:**

Our search initially identified 820 papers according to targeted keywords, out of which 65 (7.9%) papers met our criteria and were included in the review. The most popular conversational LLM was ChatGPT (60/65, 92% of papers), followed by Bard (Google LLC; 1/65, 2% of papers), LLaMA (Meta; 1/65, 2% of papers), and other LLMs (6/65, 9% papers). These papers were classified into four categories of applications: (1) summarization, (2) medical knowledge inquiry, (3) prediction (eg, diagnosis, treatment recommendation, and drug synergy), and (4) administration (eg, documentation and information collection), and four categories of concerns: (1) reliability (eg, training data quality, accuracy, interpretability, and consistency in responses), (2) bias, (3) privacy, and (4) public acceptability. There were 49 (75%) papers using LLMs for either summarization or medical knowledge inquiry, or both, and there are 58 (89%) papers expressing concerns about either reliability or bias, or both. We found that conversational LLMs exhibited promising results in summarization and providing general medical knowledge to patients with a relatively high accuracy. However, conversational LLMs such as ChatGPT are not always able to provide reliable answers to complex health-related tasks (eg, diagnosis) that require specialized domain expertise. While bias or privacy issues are often noted as concerns, no experiments in our reviewed papers thoughtfully examined how conversational LLMs lead to these issues in health care research.

**Conclusions:**

Future studies should focus on improving the reliability of LLM applications in complex health-related tasks, as well as investigating the mechanisms of how LLM applications bring bias and privacy issues. Considering the vast accessibility of LLMs, legal, social, and technical efforts are all needed to address concerns about LLMs to promote, improve, and regularize the application of LLMs in health care.

## Introduction

### Background

Since ChatGPT (OpenAI) was released on November 30, 2022, extensive attention has been drawn to generative artificial intelligence (AI) and large language models (LLMs) [1]. ChatGPT is a representative conversational LLM that generates text based on its training on an extremely large amount of data from mostly the public domain [1]. Modern LLMs (such as GPT-4) incorporate in-text learning, which enables them to interpret and generalize user inputs in the form of natural language prompts that require little to no fine-tuning [2]. These LLMs have surpassed their non–transformer-based counterparts and are now capable of performing various complex natural language processing tasks, including translation and question-answering [3]. In comparison with traditional chatbots, the current array of conversational LLMs can generate seemingly human-like coherent texts [3]. Moreover, because these models are trained on publications from digital libraries, such as Common Crawl and Wikipedia, they can generate seemingly scientific and competent answers [4].

Due to the high quality of their responses and the broad training database of modern LLMs, a growing body of studies has emerged regarding the applications of chatbots, particularly ChatGPT, in the domain of health and medicine [5]. However, most LLMs are not specially designed for health care, and therefore, certain practical pitfalls may exist when they are put into practice in that setting. Thus, there is a need to compile the latest achievements in this domain so that potential issues and guidance for new research directions can be laid out. Several reviews have been published to discuss the appropriateness of a particular application of LLMs in a specific aspect [1,6-9] but none of them summarized the overall problems systematically [8]. For example, Huang et al [6] and Giannakopoulos et al [10] summarized the application of ChatGPT only in dentistry without considering the broader landscape of other subfields in health care. Wang et al [7] discussed the ethical considerations of using ChatGPT in health care; they did not consider other LLMs for analysis, account for other common challenges, such as reliability, or mention detailed applications of the models. Moreover, their work focused on LLMs’ educational and research applications rather than their clinical use. Although Sallam [8] conducted a systematic review, the articles considered in the review were mostly editorials, letters to the editors, opinions, commentaries, news articles, and preprints, as opposed to research articles. In addition, Sallam [8] focused on educational and research applications of ChatGPT only. Puladi et al [11] narratively reviewed papers on the applications of LLMs in oral and maxillofacial surgery. Pool et al [12] reviewed papers on the application of LLMs in telehealth. Park et al [9] conducted a scoping review of papers on the medical applications of LLMs. These papers are limited in either focusing on a specific medical application area, including nonpeer-reviewed articles, or lacking a systematic examination of the concerns regarding conversational LLMs.

### This Review

This review focuses on peer-reviewed research articles on conversational LLMs that emerged after ChatGPT, which was initially based on GPT-3 (OpenAI), and their applications in health care. We aim to summarize the applications of conversational LLMs in the field of health care with concrete applications and identify potential concerns about the use of such LLMs in this field that need to be addressed in the future.

## Methods

### Search Strategy

We searched for articles that contained at least 1 word associated with LLMs (“ChatGPT,” “LLaMA,” “GPT-3,” “LaMDA,” “PalM,” “MT-NLG,” “GATO,” “BLOOM,” “Alpaca,” “Large Language Model”) and at least 1 word associated with health care (“health,” “diagnosis,” “intervention,” “patient”) published before September 1, 2023, on PubMed, ACM Digital Library, and IEEE Xplore. This systematic review applied the PRISMA (Preferred Reporting Items for Systematic Reviews and Meta-Analyses) guidelines (Multimedia Appendix 1) to steer the literature search [13-15]. Relevant publications were gathered and downloaded on September 3, 2023. For simplicity, all the LLMs mentioned henceforth refer to conversational LLMs.

### Criteria

Textbox 1 summarizes the inclusion and exclusion criteria for articles. Specifically, the inclusion criteria for a paper were as follows: (1) it was published as a peer-reviewed scientific research article between November 1, 2022, and September 1, 2023, and (2) it focuses on applications of LLMs in addressing a health care–related problem, which includes, but is not limited to, promotion of personal or public health and well-being or the potential to alleviate the workload of health care providers. We excluded a paper if it was (1) not a peer-reviewed research article, (2) not related to health care applications (eg, LLMs applied to preparing manuscripts for peer review), (3) not accessible, (4) a duplicate of an existing paper, or (5) about LLMs released before GPT-3, such as bidirectional encoder representations from transformers (BERT). We excluded BERT-related papers because this LLM, which was built upon the encoder of a transformer, is mainly applied in fine-tuning downstream machine-learning tasks. While the implementation of a chatbot based on BERT is feasible, it waned in popularity as an LLM after the introduction of ChatGPT, which was built upon the decoder of a transformer. The complete set of papers meeting the criteria were downloaded from the 3 digital libraries for further screening. Specifically, 5 of the authors of this review (LW, ZW, CN, QS, and YL) participated in paper screening and summarization under the supervision of the corresponding author, ZY. A screening protocol was created collectively after the team jointly reviewed 50 randomly selected papers. Each unreviewed paper was then screened by not fewer than 2 authors based on the protocol. All the papers in the final collection were summarized by the coauthors according to their LLM applications in health care and the concerns raised.

**Paper inclusion and exclusion criteria.**

**Inclusion criteria**

- Article type: peer-reviewed scientific research article
- Written language: English
- Time of publications: published between November 1, 2022, and September 1, 2023
- Accessibility: accessible
- Duplication: is not a duplicate of an existing article
- Models: conservational large language models (LLMs) after GPT-3 was launched
- Topic: any topics related to health care, which includes, but is not limited to, promotion of personal or public health and well-being or the potential to alleviate the workload of health care providers

**Exclusion criteria**

- Article type: any other types of publications
- Written language: any non-English language
- Time of publications: published before November 1, 2022, or after September 1, 2023
- Accessibility: not accessible
- Duplication: is a duplicate of an existing article
- Models: LLMs before GPT-3 was launched or not used for conversations
- Topic: any other topics that are not related to health care applications (eg, preparing manuscripts for peer review)

## Results

### Overview

**Figure 1:**
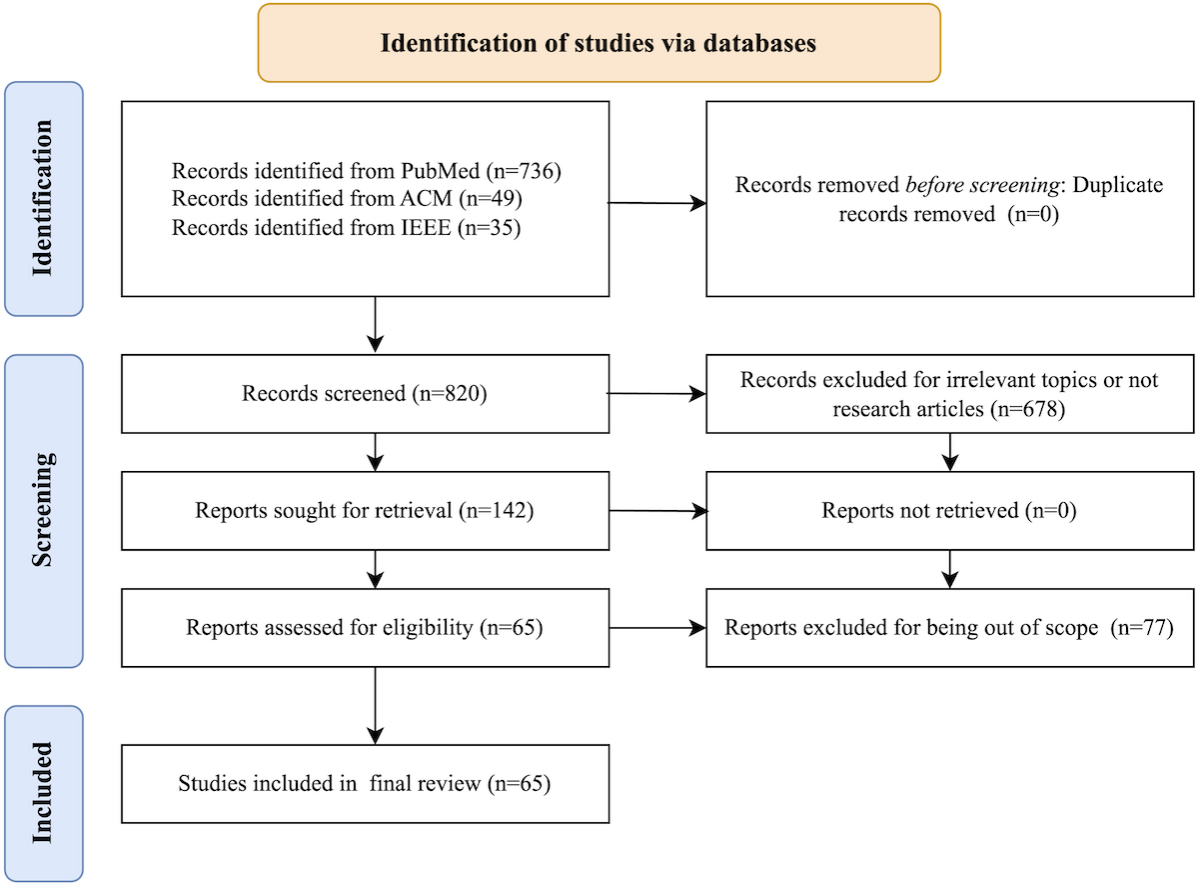
A flowchart of the article selection process based on the PRISMA (Preferred Reporting Items for Systematic Reviews and Meta-Analyses) guidelines.

Figure 1 demonstrates the paper selection process. The initial keyword search identified 820 articles, with 736 (89.8%) articles from PubMed, 49 (6%) papers from ACM Digital Library, and 35 (4.3%) papers from IEEE Xplore. The evaluation of the 820 articles was distributed among the authors for screening the titles and abstracts. The interrater reliability was assessed by computing a κ score, yielding a value of 0.72. After screening, we excluded 599 (81.4%) of the 736 articles from PubMed, 46 (94%) of the 49 articles from ACM Digital Library, and 33 (94%) of the 35 papers from IEEE Xplore because they were either not relevant to the research topic or were not research articles. No duplicates were found after the screening. Next, we extracted the full papers for the remaining 142 (17.3%) of 820 research articles and manually examined them for the 5 exclusion criteria (refer to the Methods section). This led to a final set of 65 (7.9%) of 820 articles for full-paper review and summarization—63 (97%), 2 (3%), and 0 from PubMed, ACM Digital Library, and IEEE Xplore, respectively. Among these selected articles, 60 (92%) were related to ChatGPT, 1 (2%) was related to LLaMA (Meta), 1 (2%) was related to Bard based on Language Model for Dialogue Applications (Google LLC), and 6 (9%) were related to other LLMs (Table 1 lists the specific LLM or LLMs mentioned in each selected paper). Note that 2 selected papers were related to >1 LLM, respectively.

**Figure 2:**
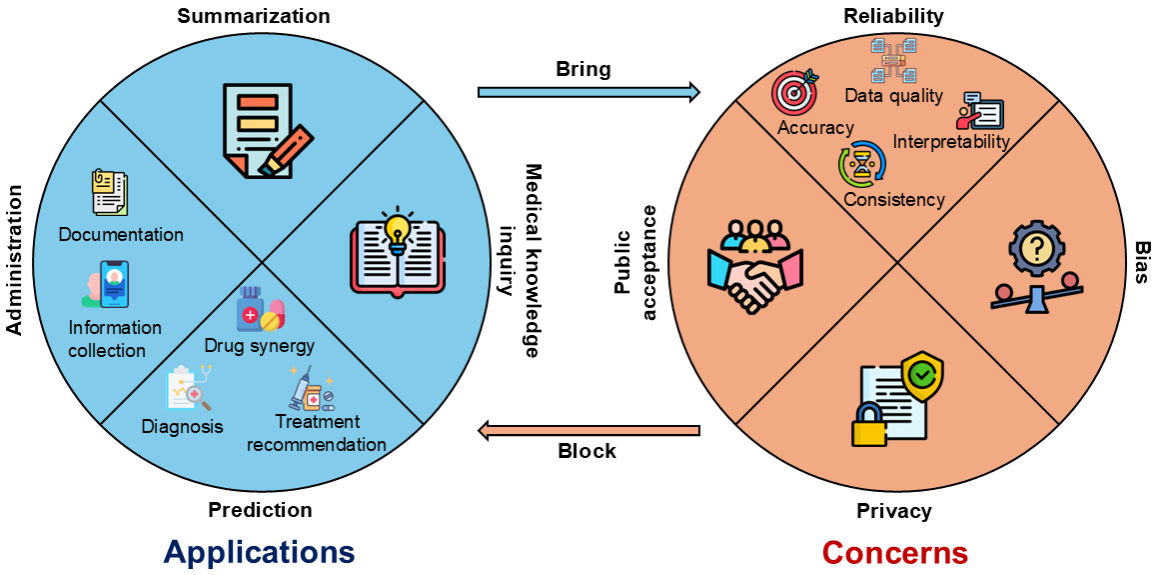
A summary of the applications and concerns about large language models (LLMs) in health care as communicated by the reviewed papers.

Five of the authors (LW, ZW, CN, QS, and YL) compiled the topics related to applications and concerns independently during the paper screening and summarization process. Furthermore, through extensive discussions, all the authors refined and categorized these topics into main applications and concerns with corresponding subcategories. Figure 2 illustrates the main topics of applications and concerns mentioned by the reviewed papers on applying LLMs in health care settings. The multifaceted applications of LLMs can be divided into 4 primary categories: summarization, medical knowledge inquiry, prediction, and administration: summarization (25/65, 38% papers)—LLMs are potential tools for summarizing complex information or documentation in clinical domains. Medical knowledge inquiry (30/65, 46% papers)—LLMs demonstrate proficiency in answering a diverse array of medical questions and examinations, which enhance public access to medical knowledge. Prediction (22/65, 34% papers)—LLMs demonstrate high *diagnostic* accuracy in multiple medical scenarios (15/65, 23% papers), offer assistance in diverse *treatments* (12/65, 18% papers), and excel in predicting drug interactions and *synergies* (1/65, 2% paper). Administration (9/65, 14% papers)—LLMs streamline various tasks, including *documentation* (5/65, 8% papers) and *information collection* (5/65, 8% papers) to monitor the trend of public health.

The concerns surrounding the application of LLMs in health care were varied, each with nuanced considerations: Reliability (55/65, 85% papers)—This includes *accuracy* (45/65, 69% papers), or the correctness of the responses from LLMs; *consistency* (13/65, 20% papers), whether LLMs produce the same response to the same questions with different prompts; *interpretability* (5/65, 8% papers), whether LLMs can explain their responses well, and the data *quality* of the training dataset (16/65, 25% papers). Bias (16/65, 25% papers)—The applications of LLMs may result in biased responses, which will exacerbate disparity and inequality in health care, particularly in terms of *financial costs* (1/65, 2% paper), *readability* (5/65, 8% papers), and *accessibility* (3/65, 5% papers). Privacy (6/65, 9% papers)—Training LLMs in health care settings requires a large number of health data which, however, is sensitive and may bring privacy issues.

Public acceptance (4/65, 6% papers): Building trust in LLMs from the public is pivotal for widespread acceptance and use of LLM-based health care applications.

**Table 1:** A summary of applications and concerns of the reviewed research papers. Subcategories of a paper are shown in parentheses immediately after the paper’s category.

Reference	Application		Concern		LLMs^a^	Authors’ countries
	Category	Note	Category	Note		
Akhter and Cooper [16]	Summarization	The authors used ChatGPT to generate a case report for a patient who developed a common complication.	Reliability (accuracy and data quality: data timeliness)	This paper shows that ChatGPT sometimes cites nonexistent sources and is currently limited in critically discussing results and literature.	ChatGPT	United States
Almazyad et al [17]	Summarization	This paper shows that ChatGPT can summarize conference medical recommendations.	Reliability (accuracy) and bias (biased training data)	This paper shows that results from ChatGPT can be biased.	ChatGPT-4	Saudi Arabia and Lebanon
Bhattacharyya et al [18]	Summarization	The authors investigated the authenticity and accuracy of references in ChatGPT-generated medical articles.	Reliability (accuracy)	This paper shows that ChatGPT often generates fabricated or inaccurate medical references, with a high prevalence of errors in reference elements.	ChatGPT-3.5	United States
Bosbach et al [19]	Summarization	In this paper, ChatGPT drafts competent radiology reports with high appraisal given command files as input.	Reliability (accuracy)	In this paper, ChatGPT showed limitations in its ability to deal with technical or medical terminology.	ChatGPT	Switzerland, Germany, Poland, Hungary, and Malaysia
Chen et al [20]	Summarization	In this paper, ChatGPT is used to construct a risk factor database for diseases, demonstrating its potential to extract data from literature abstracts effectively.	Reliability (accuracy)	This paper discusses the challenges in accurately extracting risk factor information, emphasizing the need for human validation to ensure model accuracy.	ChatGPT	China
Lahat et al [21]	Summarization	This paper discusses ChatGPT’s potential in formulating gastroenterology research questions.	—^b^	—	ChatGPT	Israel and the United States
Puthenpura et al [22]	Summarization	The case report uses ChatGPT to integrate AI^c^-generated text with original author writing.	Reliability (interpretability and accuracy)	This paper notes the potential for AI-generated text to be inaccurate and provides nonexistent references in information. Dependence on AI tools may lead to overlooking subtle clinical signs by clinicians.	ChatGPT	United States
Robinson and Aggarwal [23]	Summarization	ChatGPT’s effectiveness in drafting operation notes for appendicectomy is evaluated in this paper, demonstrating adherence to NHS^d^ surgical documentation guidelines.	Reliability (consistency)	This paper notes the dependency of ChatGPT’s output quality on the prompt and emphasizes the need for secure integration with health records for surgical documentation.	ChatGPT	United Kingdom
Zhou [24]	Summarization	The authors explored ChatGPT’s capabilities in generating medical reports from laboratory results with the goal of streamlining the report generation process.	Reliability (accuracy)	This paper raises concerns about the precision and reliability of medical report generation by ChatGPT.	ChatGPT	United States
Guirguis et al [25]	Summarization	The authors used ChatGPT to generate a case report for a patient with neurosarcoidosis.	—	—	ChatGPT	United States
Haemmerli et al [26]	Summarization (case report) and prediction (diagnosis, treatment recommendation)	The authors evaluated ChatGPT’s performance in brain glioma adjuvant therapy decision-making. It can enhance medical case reporting and study data analysis for manuscript production.	Reliability (accuracy, data quality: data timeliness) and bias (biased algorithm)	This paper shows that ChatGPT is less accurate and more biased in the interpretation of medical information. It also shows that ChatGPT lacks live internet access and access to research databases.	ChatGPT	Switzerland, Lithuania, Austria, and the United States
Lyu [27]	Summarization (clinical notes)	This paper shows ChatGPT’s ability to translate radiology reports into plain language with nice results.	Reliability (accuracy and consistency)	This paper shows concerns regarding ChatGPT’s moral and legal issues. For example, ChatGPT tends to oversimplify or overlook and omit key points during translation, resulting in inaccuracy.	ChatGPT, GPT-4	United States
Cunningham et al [28]	Summarization and medical knowledge inquiry	This paper uses ChatGPT to aid in manuscript synthesis of a patient with glioblastoma in the pineal gland exhibited over 5 years of survival following radiotherapy and temozolomide.	Reliability (accuracy, data quality: date timeliness, and interpretability) and bias (readability)	This paper shows that ChatGPT cannot substitute professional medical advice and has concerns about its knowledge cutoff of 2021 and its inability to access the internet.	ChatGPT	United States
Golan et al [29]	Summarization and medical knowledge inquiry	The authors used ChatGPT to evaluate the quality and readability of online medical text regarding shock-wave therapy for erectile dysfunction	Reliability (accuracy) and bias (readability)	This paper shows that ChatGPT, in its current state, is less effective than human reviewers and a reliable tool [30].	ChatGPT	United States
Hamed et al [31]	Summarization and medical knowledge inquiry	This study integrates ChatGPT-4 with “Link Reader” for automating medical text synthesis, improving AI models’ traceability and retrieval accuracy.	—	—	ChatGPT-4	Qatar and the United Kingdom
Grewal et al [32]	Summarization, medical knowledge inquiry, and prediction (treatment recommendation)	This paper highlights ChatGPT’s applications in radiology, including report generation, template creation, patient communication, clinical decision-making enhancement, research title suggestion, scholarly article heading creation, and formatting and referencing for research papers.	Reliability (data quality: data source and data timeliness) and privacy	This paper shows that ChatGPT is limited by biases in its training data, may produce inaccuracies, and integrating it with EHRs^e^ risks. It may further be hampered by outdated training data.	ChatGPT (GPT-4)	United States and India
Kumari and Anusha [33]	Summarization, medical knowledge inquiry, and prediction (treatment recommendation)	This paper incorporates information from ChatGPT in the treatment planning and case report writing.	Reliability (accuracy) and public acceptance	This paper shows that ChatGPT provides more general information.	ChatGPT	India
Cadamuro et al [34]	Summarization and prediction (diagnosis)	This paper evaluates ChatGPT with laboratory reports for relevance, correctness, helpfulness, and safety, suggesting that it can interpret individual tests but not an overall diagnostic picture.	Reliability (accuracy and interpretability)	In this paper, ChatGPT incorrectly interpreted normal results for suspected diseases and struggled to synthesize all related laboratory test findings coherently.	ChatGPT	Austria, Italy, Croatia, Belgium, Spain, and Turkey
Jiang et al [35]	Summarization and prediction (treatment recommendation)	The authors developed NYUTron, a LLM trained on unstructured clinical notes for clinical predictive tasks.	Reliability (accuracy and consistency) and bias (accessibility)	This paper shows that it is hard to ensure the accuracy and reliability of predictions in a clinical setting. The model lacks generalizability.	NYUTron	United States, Canada
Sharma et al [36]	Summarization and prediction (diagnosis)	The authors developed DR.BENCH^f^, a generative AI framework for clinical diagnostic reasoning tasks. They showed that a multitask, clinically trained language model significantly outperforms general domain models.	—	—	DR.BENCH	United States
Liu et al [37]	Summarization, prediction (diagnosis and treatment recommendation), medical knowledge inquiry, and administration (documentation)	The authors explored ChatGPT’s roles in clinical practice, focusing on clinical decision support, question-answering, and medical documentation.	Reliability (accuracy) and privacy	The authors pointed out that potential negatives such as privacy, ethics, bias, and discrimination, compounded by outdated training data, cannot be overlooked.	ChatGPT	China, United States
Hamed et al [38]	Summarization (clinical notes) and administration (documentation)	This paper demonstrates ChatGPT’s ability to adapt clinical guidelines.	—	—	ChatGPT	Qatar, United Kingdom
Kim [39]	Summarization and administration (documentation)	This paper introduces a case study that shows that ChatGPT can help with medical documentation.	—	—	ChatGPT	United States, Korea
Macdonald et al [40]	Summarization and administration (documentation)	This paper demonstrates that ChatGPT can write a paper giving a dataset.	Reliability (accuracy)	The authors pointed out that ChatGPT can produce incorrect references and pass plagiarism detectors with a 100% score.	ChatGPT	United Kingdom
Cascella et al [41]	Summarization and administration (documentation and information collection)	This paper shows that ChatGPT can summarize information, list possible research topics, and write clinical notes.	—	—	ChatGPT	Italy
Ali [42]	Medical knowledge inquiry	This paper shows that ChatGPT can provide information about lacrimal drainage disorders.	Reliability (accuracy)	This paper shows that the information from ChatGPT is not all correct.	ChatGPT	India
Antaki et al [43]	Medical knowledge inquiry	This paper evaluates ChatGPT’s proficiency in answering ophthalmic questions, showing promising results in a simulated OKAP^g^ examination.	Reliability (accuracy and data quality: data source)	This paper shows that ChatGPT’s accuracy depends on concordance and insight, with inaccuracies often due to insufficient training.	ChatGPT, ChatGPT plus	Canada
Bird and Lotfi [44]	Medical knowledge inquiry	The authors optimized a chatbot that can answer questions regarding mental health with high accuracy.	Reliability (data quality: data source)	The authors pointed out that the available data are limited, and it takes a lot of efforts to collect data.	An unnamed chatbot	United Kingdom
Hoch et al [45]	Medical knowledge inquiry	This paper shows that ChatGPT displays high quiz skills and accuracy in examinations.	Reliability (accuracy)	This paper shows that ChatGPT can give false answers to a substantial proportion of questions in specific otolaryngology subdomains.	ChatGPT	Germany, United States, Spain, and the United Kingdom
Holmes et al [46]	Medical knowledge inquiry	In this paper, LLMs, including ChatGPT, are evaluated on radiation oncology physics, with GPT-4 exhibiting superior performance and reasoning abilities.	Reliability (consistency and accuracy)	This paper highlights ChatGPT’s consistency in answering radiation oncology physics questions yet underscores the superior performance of a team of medical physicists.	ChatGPT (GPT-3.5), ChatGPT (GPT-4), Bard (LaMDA^h^), and BLOOMZ	United States
Hristidis et al [47]	Medical knowledge inquiry	In this paper, ChatGPT and Google are compared for dementia-related queries, assessing the quality and reliability of their responses.	Bias (readability)	This paper comments on the relevance and readability of responses from ChatGPT and Google for dementia-related queries, noting challenges in both platforms.	ChatGPT	United States
Johnson et al [48]	Medical knowledge inquiry	The authors assessed ChatGPT’s ability to answer cancer information-related questions, indicating that ChatGPT provides accurate information about common cancer myths and misconceptions.	Reliability (accuracy, data quality: data timeliness) and bias (biased algorithm)	The authors advocated that future evaluation of AI platforms needs infrastructure to monitor for bias and health disparities, considering user trust and credibility in AI responses.	ChatGPT	United States
Kung et al [49]	Medical knowledge inquiry	This paper investigates ChatGPT’s capability to surpass USMLE’s^i^ passing threshold, showing its increasing accuracy and potential in medical education.	Reliability (accuracy and consistency)	This paper shows that AI’s performance in medical examinations limited to human perception.	ChatGPT	United States
Kusunose et al [50]	Medical knowledge inquiry	In this paper, ChatGPT provided accurate responses to CQs^j^ related to the JSH^k^ 2019 guidelines for the management of hypertension.	Reliability (accuracy)	In this paper, ChatGPT did not provide accurate responses to some questions.	ChatGPT	Japan
Lahat et al [51]	Medical knowledge inquiry	Evaluating ChatGPT’s answers to gastrointestinal health questions, this study indicates its capacity to provide accurate information in certain areas.	Reliability (accuracy)	This paper highlights the varying quality of ChatGPT’s information and emphasizes the need for further development to enhance its utility for patients.	ChatGPT	Israel and the United States
Li et al [52]	Medical knowledge inquiry	The authors refined LLaMA using 100,000 patient-doctor dialogues to provide medical advice.	Reliability (accuracy)	The paper shows that the accuracy of LLMs such as ChatGPT could be significantly improved if they could generate or assess responses based on a reliable knowledge database with experiments.	LLaMA	United States and China
Moshirfar et al [53]	Medical knowledge inquiry	GPT-4 outperforms GPT-3.5 and human experts in answering ophthalmology questions, with significant variations across different difficulty levels.	Reliability (accuracy, data quality: data timeliness) and bias (financial costs and accessibility)	The drawbacks of using GPT-4 include paying a monthly fee and having a knowledge cutoff of September 2021.	ChatGPT (GPT-3.5) and ChatGPT (GPT-4)	United States
Nov et al [54]	Medical knowledge inquiry	This paper assesses ChatGPT’s answers to patient questions with health care providers, indicating its effectiveness in generating patient responses.	Reliability (accuracy, data quality: data source), public acceptance, and bias (biased training data)	This paper shows that ChatGPT and similar LLMs face issues such as biased or incorrect responses, with automation bias and liability concerns requiring vigilant chatbot response curation.	ChatGPT	United States
Sallam et al [55]	Medical knowledge inquiry	This paper shows that ChatGPT can challenge misinformation, such as COVID-19 vaccine conspiracies.	Reliability (accuracy) and bias (biased training data)	The authors pointed out that ChatGPT only has limited knowledge by 2021, so it is possible that it can produce biased and unreliable results.	ChatGPT	Jordan, Lebanon, and Indonesia
Sinha et al [56]	Medical knowledge inquiry	This paper shows the high accuracy of ChatGPT to solve higher-order reasoning questions in pathology.	Reliability (accuracy, data quality: data timeliness)	The authors pointed out that ChatGPT has limitations in that they have information on 2021, and future AI systems must be carefully designed, developed, and validated to ensure they provide accurate information.	ChatGPT	India
Thirunavukarasu et al [57]	Medical knowledge inquiry	This paper assesses ChatGPT’s primary care application, showing promise but necessitating further development as indicated by its AKT^l^ performance.	Reliability (accuracy) and public acceptance	This paper acknowledges the potential and current limitations of ChatGPT in primary care, indicating a need for further development to reach the expertise level of qualified physicians.	ChatGPT	United Kingdom
Van Bulck and Moons [58]	Medical knowledge inquiry	In this paper, 17 of 20 experts consider ChatGPT provides answers of a higher or equal value compared with Google search.	Reliability (consistency)	This paper shows that ChatGPT is sensitive to nuance in the prompts. It uses outdated training data and is less transparent with its sources.	ChatGPT	Belgium, Sweden, and South Africa
Wagner and Ertl-Wagner [59]	Medical knowledge inquiry	The accuracy of ChatGPT-3 in retrieving clinical radiological information is tested in this paper, cross-checking its responses with peer-reviewed references.	Reliability (accuracy)	This paper expresses concerns about the accuracy and authenticity of ChatGPT-3’s radiological information and references.	ChatGPT-3	Canada
Walker et al [60]	Medical knowledge inquiry	This study evaluates the reliability of medical information from ChatGPT-4 using the EQIP^m^ tool and comparison with clinical guidelines for 5 hepato-pancreaticobiliary conditions.	Reliability (accuracy, consistency, and interpretability) and bias (readability)	This paper shows that ChatGPT-4 has no support for references, complicated answers, and accuracy issues.	ChatGPT-4	United Kingdom, Switzerland, and Saudi Arabia
Yeo et al [61]	Medical knowledge inquiry	The performance of ChatGPT in responding to questions about cirrhosis and hepatocellular carcinoma is assessed in this paper, showing extensive knowledge in these areas.	Reliability (data quality)	This paper expresses concerns about ChatGPT’s limitations in providing comprehensive and region-specific knowledge, particularly in managing cirrhosis and hepatocellular carcinoma.	ChatGPT	United States, United Kingdom
Zhu et al [62]	Medical knowledge inquiry	This paper shows that ChatGPT is able to pass the Chinese Medical Licensing Examination’s Clinical Knowledge Section.	Reliability (accuracy) and privacy	The authors pointed out the importance of privacy, accuracy, and reliability for an AI system.	ChatGPT	China
Altamimi et al [63]	Medical knowledge inquiry and prediction (treatment recommendation)	In this study, ChatGPT is assessed for providing advice on venomous snakebites and offering accurate management information in simulated consultations.	Reliability (accuracy)	This paper discusses reliability concerns of ChatGPT in providing snakebite management advice, stressing the need for updated knowledge and personalized information.	ChatGPT	Saudi Arabia
Goktas et al [64]	Medical knowledge inquiry and prediction (diagnosis)	The authors gave examples of using “ChatGPT 4.0” in the field of allergy and immunology.	Reliability (accuracy), bias, and privacy	The authors pointed out the importance of privacy and reliability.	ChatGPT 4.0	Ireland, Turkey
Chervenak et al [65]	Medical knowledge inquiry and administration (information collection)	This paper evaluates ChatGPT’s performance on fertility-related clinical queries.	Reliability (accuracy)	This paper shows that: (1) ChatGPT provides an illusion of reliability in its persuasive prose. (2) Different patient populations may interact with ChatGPT in different ways. (3) ChatGPT is not able to reliably cite sources.	ChatGPT	United States
Agbavor and Liang [66]	Prediction (diagnosis)	This research shows GPT-3-based text embeddings can differentiate patients with Alzheimer from healthy controls through speech data, suggesting early diagnostic potential.	Reliability (accuracy)	This paper acknowledges the limited research on using LLMs for early dementia diagnosis, specifically the potential of GPT-3.	GPT-3	United States
Hirosawa et al [4]	Prediction (diagnosis)	This paper shows that ChatGPT-3 can generate a diagnosis list for common chief complaints with high accuracy.	Reliability (data quality: data source, interpretability) bias (biased training data)	The papers show that it is unclear about the hyperparameters and training algorithms of the ChatGPT, thus it lacks transparency or interpretability. In addition, ChatGPT may produce misleading and biased results. Last, ChatGPT lacks recent knowledge.	ChatGPT-3	Japan
Huang et al [6]	Prediction (diagnosis)	This study shows that ChatGPT can be used in dental diagnosis.	Reliability (accuracy), bias (accessibility), and privacy	This study shows that ChatGPT may violate patients’ privacy, and ChatGPT cannot truly understand data and may produce biased results.	ChatGPT	China and United States
Karkera et al [67]	Prediction (diagnosis)	The authors assessed various pretrained LLMs for extracting microbe-disease relationships from biomedical texts in zero-shot or few-shot contexts.	Reliability (consistency) and bias (algorithm bias)	This paper shows that varying outputs for identical prompts raise concerns about the model’s response reliability.	GPT-3, BioGPT, BioMedLM, BioMegatron, PubMedBERT, BioClinicalBERT, and BioLinkBERT	Japan, India
Sarbay et al [68]	Prediction (diagnosis)	ChatGPT’s performance in emergency triage prediction is assessed in this paper, comparing its predictions with expert categories and scoring its sensitivity and specificity.	Reliability (accuracy)	This paper notes discrepancies and inconsistencies in some cases.	ChatGPT	Turkey
Shahsavar and Choudhury [3]	Prediction (diagnosis)	This paper examines factors that influence users’ intentions to use ChatGPT for self-diagnosis and health-related purposes, revealing a high willingness to adopt the technology.	Reliability (data quality) and public acceptance	The paper notes that ChatGPT is not specifically designed for health care purposes, which may affect its suitability for self-diagnosis.	ChatGPT	United States
Galido et al [69]	Prediction (diagnosis and treatment recommendation)	This paper shows that ChatGPT can identify patients as having TRS^n^ accurately, make treatment suggestions, and identify drug side effects in the treatment recommendation.	Reliability (consistency and data quality: data source)	This paper shows that ChatGPT can be combined with commercial applications while generating answers. However, its output is influenced by incorrect input, and it lacks clinical context and the ability to request edits to input errors.	ChatGPT	United States
Sorin et al [70]	Prediction (diagnosis, treatment recommendation)	In this paper, ChatGPT is evaluated as a decision support tool for the breast tumor board, showing promise in recommending management aligned with tumor board decisions.	Reliability (accuracy and consistency)	This paper reflects on the alignment of ChatGPT with tumor board recommendations, showing potential as a decision support tool with a 70% concordance rate.	ChatGPT	Israel
Juhi et al [71]	Prediction (drug synergy)	In this paper, ChatGPT is tested on predicting and explaining drug-drug interactions, aiming to enhance patient safety by providing accurate drug compatibility information.	Reliability (accuracy)	This paper shows that ChatGPT sometimes provides incomplete information.	ChatGPT	India
Haver et al [72]	Prediction (treatment recommendation)	In this paper, ChatGPT demonstrates high accuracy in providing recommendations and prevention of breast cancer.	Reliability (consistency)	This paper shows that ChatGPT is sensitive to nuance in the prompts. ChatGPT is a research “chatbot” not specially designed for medical use.	ChatGPT	United States
Liu et al [73]	Prediction (treatment recommendation)	The authors compared ChatGPT-generated clinical support alerts with human-made suggestions, highlighting its potential for unique, understandable, and relevant contributions.	Reliability (consistency, accuracy, and data quality: data timeliness)	The authors showed that ChatGPT’s responses vary with prompt changes, highlighting its sensitivity to different input sentences.	ChatGPT	United States
Kao et al [74]	Prediction (treatment recommendation, diagnosis)	The authors evaluated ChatGPT as a CDS^o^ tool in pediatrics, suggesting its capability to improve clinical workflow and assist in responsible decision-making.	Reliability (accuracy)	The authors argued that AI technologies, such as ChatGPT, are not yet advanced enough to replace doctors in complex diagnoses or treatment planning.	ChatGPT	China
Schulte [75]	Prediction (treatment recommendation and diagnosis)	ChatGPT was used to identify guideline-based treatments for advanced solid tumors with a VTQ^p^ of 0.77 when being compared with NCCN^q^ guidelines.	Reliability (accuracy and consistency)	The authors showed that ChatGPT’s accuracy and consistency are not certain.	ChatGPT	United States
Carpenter and Altman [76]	Administration (information collection)	In this paper, GPT-3 is used to generate a drug abuse lexicon from social media slang, aiming to improve pharmacovigilance and monitor of drug abuse trends.	Reliability (accuracy) and bias (readability)	This paper acknowledges challenges in generating a reliable lexicon for drug abuse synonyms due to the variability of social media language.	GPT-3	United States
Jo et al [2]	Administration (information collection)	The authors built CareCall, an AI tool built on HyperCLOVA, which aims at monitoring the health conditions of socially isolated groups.	Reliability (data quality: data source)	The authors pointed out that firsthand data are hard to gather because collecting personal health data may give rise to privacy issues.	CareCall	United States and Korea
Montagna et al [77]	Administration (information collection)	The authors used GPT-3 to develop an LLM-based chatbot to support management of patients’ health data related to chronic diseases.	Reliability (data quality: data source) and privacy	The paper shows that LLMs lack medical expertise and may be influenced by any bias in the data they were trained on. How to protect patients’ privacy is another issue the authors pointed out in this paper.	GPT-3	Italy

^a^LLM: large language model.

^b^Not available.

^c^AI: artificial intelligence.

^d^NHS: National Health Service.

^e^EHR: electronic health record.

^f^DR.BENCH: Diagnostic Reasoning Benchmark.

^g^OKAP: Ophthalmic Knowledge Assessment Program.

^h^LaMDA: Language Model for Dialogue Applications.

^i^USMLE: United States Medical Licensing Exam.

^j^CQ: clinical question.

^k^JSH: Japanese Society of Hypertension.

^l^AKT: Applied Knowledge Test.

^m^EQIP: Ensuring Quality Information for Patients.

^n^TRS: treatment-resistant schizophrenia.

^o^CDS: clinical decision support.

^p^VTQ: valid therapy quotient.

^q^NCCN: National Comprehensive Cancer Network.

### Applications

All reviewed research papers demonstrated the usability or tested the capability of LLMs for health care applications in clinical or research domains, which can be further classified into the following 4 categories: summarization, medical knowledge inquiry, prediction, and administration.

#### Summarization

ChatGPT has been shown to be effective in summarizing medical documents for a diverse set of applications [37,39], including tasks such as adapting clinical guidelines for diagnosis, treatment, and disease management [38], summarizing medical notes [19,23,41], assisting in writing medical case reports [16,22,24-26,35], and generating and translating radiological reports [19,32]. Notably, efforts have been made to integrate ChatGPT-4 with the Link Reader plugin for automating medical text synthesis [31], which boosted model performance in providing answers according to clinical guidelines [31]. Another study by Zhou [24] explored ChatGPT’s role in supporting health care professionals in creating medical reports from real patient laboratory results to offer treatment recommendations based on patients’ health conditions [24].

ChatGPT proved beneficial for summarizing research papers as well [17]. Notably, it demonstrated impressive performance in summarizing conference panels and recommendations [17], generating research questions [21], extracting data from literature abstracts [20], drafting medical papers based on given datasets [40], and generating references from medical articles [18]. ChatGPT was also used to evaluate the quality and readability of web-based medical text regarding shock-wave therapy for erectile dysfunction [29]. These applications highlighted the potential of LLMs to condense complex and extensive research materials, allowing for more accessible comprehension and use of information in health care.

#### Medical Knowledge Inquiry

ChatGPT can be applied to answer questions about health care, as evidenced by its excellent performance in various studies [28,31,42,50,51,53,54,59-61,63]. For instance, ChatGPT has shown remarkable accuracy in reasoning questions and medical exams [45,56], even successfully passing the Chinese Medical Licensing Examination [62] and the United States Medical Licensing Examination [49]. It also performed well in addressing radiation oncology physics examination questions [46]. Likewise, “ChatGPT would have been at the 87th percentile of Bunting’s 2013 international cohort for the Cardiff Fertility Knowledge Scale and at the 95th percentile on the basis of Kudesia’s 2017 cohort for the Fertility and Infertility Treatment Knowledge Score” [65]. In addition, ChatGPT showed promising results in a simulated Ophthalmic Knowledge Assessment Program exam [43]. However, the average score of ChatGPT was 60.17% in the Membership of the Royal College of General Practitioners Applied Knowledge Test, which is <70.4%, the mean passing threshold in the last 2 years [57].

Furthermore, LLMs have been shown to be effective at making medical knowledge accessible to the public. In particular, a fine-tuned chatbot based on LLaMA demonstrated enhanced performance in identifying patients’ needs and providing informed suggestions [52]. In the realm of medical advice, ChatGPT-generated educational documents, answered questions about allergy and immunology [64], and countered vaccine conspiracy theories [55]. It can also answer the most frequently asked questions about the COVID-19 pandemic. Its overall responses to queries related to cognitive decline were equivalent to and, at times, more reliable than Google’s [47]. According to Bulck and Moons [58], in comparison with Google search, 40% (8 experts) of the 20 experts (19 nurses and 1 dietitian) considered answers from ChatGPT of greater value, 45% (9 experts) regarded them as equal value, and 15% (3 experts) deemed them less valuable. Therefore, many experts predicted that patients will gradually rely more on LLMs (particularly ChatGPT) and less on Google searches due to the high quality and accessibility of the answers from LLMs. Regarding cancer myths and misconceptions, 97% (63/65) of expert reviews deemed answers from ChatGPT to be accurate [48]. In addition, Bird and Lotfi [44] optimized a chatbot that could answer mental health–related questions with an accuracy of 88.7% (26,595/30,000 tokens) [69]. Overall, LLMs, particularly ChatGPT, demonstrate an impressive performance in public education in health.

#### Prediction

LLMs have been shown to have predictive capabilities in diagnosis, treatment recommendations, and drug interactions and synergies.

##### Diagnosis

ChatGPT has exhibited the potential to achieve high accuracy in diagnosing specific diseases [37,69], providing diagnostic suggestions in simulated situations [63,73] or using given laboratory reports for diagnosis [34]. ChatGPT has been evaluated in dental [6], allergy [64], and mental disorders diagnoses [66]. Particularly, GPT-3 can be used to differentiate patients with Alzheimer disease from healthy controls using speech data [66]. Beyond ChatGPT, other generative AI frameworks, such as DR.BENCH [36], were used for clinical diagnostic reasoning tasks [36]. Moreover, various pretrained LLMs can extract microbe-disease relationships from biomedical texts in zero-shot or few-shot contexts with high accuracy, with an average *F*_1_-score, precision, and recall >0.8 [67]. In addition, ChatGPT was the best LLM when predicting high acuity cases than predicting low acuity cases according to the emergency severity index, with a sensitivity of 0.762 and a specificity of 0.931, compared with the overall sensitivity of 0.571 and a specificity of 0.345 [68].

For example, Hirosawa et al [4] obtained ChatGPT’s diagnostic response by describing a clinical scenario. The prompt began with “Tell me the top 10 suspected illnesses for the following symptoms;” Then, patients’ personal information (eg, age and family history) was provided in this prompt along with other clinical data (eg, symptoms, medication, and physical examination). According to the study, the top 10 suspected diseases generated by ChatGPT achieved a rate of 93% (28/30) in overall correctness. While such a level of performance is impressive, physicians still made a better prediction than ChatGPT. With respect to the top 5 diagnoses, physicians achieved an accuracy of 98% (59/60) while ChatGPT only achieved 83% (25/30). As for the top suspected disease, ChatGPT only had a correct rate of 53% (16/30), versus 93% (56/60) achieved by physicians [4].

##### Treatment Recommendations

LLMs can offer treatment recommendations while listing the side effects of these treatments [69]. They have been involved in the treatment of various diseases, such as allergy and immunology [64]. ChatGPT can identify guideline-based treatments for advanced solid tumors [75], such as breast tumor treatment [70]. LLMs can also assist with treatment planning [33] and brain glioma adjuvant therapy decision-making [26]. Similarly, NYUTron, an LLM trained on unstructured clinical notes, has been applied for clinical predictive tasks in treatments [35]. ChatGPT can effectively recommend breast tumor management strategies based on clinical information from 10 patients [70], enhance clinical workflow, and assist in responsible decision-making in pediatrics [74]. In addition, ChatGPT can recommend cancer screening given the radiology reports, with an accuracy of 88% (22/25) [72]. Overall, ChatGPT performs well in certain scenarios of disease prevention and screening recommendations.

##### Drug Synergies

LLMs also demonstrate high utility when characterizing drug effects. Notably, ChatGPT was used to predict and explain drug-drug interactions [71]. In this study, the LLMs were asked about pairing or interaction between drugs, and their responses are evaluated in terms of correctness and conclusiveness. Among the 40 pairs of drug-drug interactions, 39 (98%) responses were correct for the first question, and among these 39 correct answers, 19 (49%) were conclusive while 20 (51%) were inconclusive. For the second question, 39 (97%) were correct among 40 pairs, with 17 (44%) answers conclusive and 22 (56%) answers inconclusive.

#### Administration

LLMs can serve a multifaceted role in the realm of health care and administrative tasks. Specifically, ChatGPT proves instrumental in streamlining administrative processes by generating texts, thereby alleviating the associated workload [38]. Moreover, it can be used to track patients’ health status, particularly those with chronic diseases [77]. Through the analysis of social media slang, GPT-3 aided in developing a drug abuse lexicon that was aimed at enhancing the monitoring of drug abuse trends [76]. Notably, an LLM-based chatbot, called CLOVA CareCall, built by Naver [2], was applied as a health data–collecting tool in South Korea. Designed for individuals who need emotional support and are socially isolated, CareCall conducted periodic conversations, generating health reports with metrics such as meals, sleep, and emergencies. Implemented in 20 cities by May 2022, it targeted solitary adults, notably those with lower incomes and was proven effective in reducing loneliness. Social workers used the generated reports and call recordings to monitor users’ health, resulting in positive feedback and a streamlined workload for public health workers.

### Concerns

Most of the reviewed research papers pointed out technical and ethical concerns that people harbor with respect to the application of LLMs in health care from several perspectives. This can generally be categorized into four groups: (1) reliability, (2) bias, (3) privacy, and (4) public acceptance.

#### Reliability

##### Overview

The reliability of LLMs is essential to their application in health care. It can be related to the accuracy, consistency, and interpretability of LLM responses and the quality of the training dataset. Specifically, in 100% (22/22) of prediction-related studies, 72% (18/25) of summarization-related studies, and 93% (28/30) of studies related to medical knowledge inquiries, the authors pointed out their concerns toward LLM reliability (Table 1).

##### Accuracy

Several studies highlighted that ChatGPT exhibited inaccuracies when asked to respond to certain questions [19,20,24,27,29,33,37,45,50,55,57,59,63,64,68,75,76]. For instance, ChatGPT could respond with incomplete information or exhibit an inability to distinguish between truth and falsehood [26,71]. The generative nature of the LLM algorithms will likely fabricate a fake reference to substantiate false claims [18], a process that has been referred to as “hallucinations” [73]. In addition, such hallucinations can be communicated via persuasive prose [28], making it more likely to mislead patients. For example, Jo et al [2] mentioned that LLMs (specifically CLOVA CareCall built by NAVER in this paper) may make ambitious or impractical promises to patients, which may add extra burden to therapists or cause a trust crisis [2].

##### Data Quality

The unreliability of LLMs may be attributed to limitations in data collection sources [43,69]. There are concerns about the model’s limitation in medical knowledge [61] because the general-purpose nature of ChatGPT may affect its reliability in self-diagnosis [3]. Recent state-of-the-art LLMs are typically constructed on texts from the internet rather than verified resources about health and medicine [1].

Of greater concern is the data availability. Health care institutions have shared no identifiable health information with widely accessible LLMs such as ChatGPT due to privacy concerns and legal compliances [7], and it is arduous to collect new data for LLM training [44]. ChatGPT, for example, was not trained on patients’ clinical data [4]. While a description of a clinical scenario without sensitive patient information can be fed into ChatGPT through prompts, it may lead to inaccurate responses [4].

Another contributing factor to inaccuracy is the outdated knowledge base used to train LLMs [26,32,40,53]. ChatGPT based on GPT3.5 was pretrained using data collected until 2021 and does not support internet connection [43], making it unable to perform appropriately on questions regarding events that happened after 2021 [28].

##### Consistency

Many authors expressed concerns about the inconsistency of the responses from LLMs [26,32,40], where different answers result from various prompts of the same question [23,27,29,33,54,68,69,73]. In addition, the output of ChatGPT to the same prompt may vary from user to user [23]. This is because LLMs generate responses in a probabilistic manner [1]. Therefore, nuance in the prompts to the LLM may lead to a completely different answer [23].

##### Interpretability

Interpretability is another aspect regarding the reliability of the response. A study by Cadamuro et al [34] highlights 2 key issues with an LLM (particularly ChatGPT) in health care. First, the interpretation of some normal results regarding suspected underlying diseases was not completely correct. Second, ChatGPT struggled to interpret all the coherent laboratory tests [34], generating superficial and incorrect responses. Indeed, ChatGPT could generate overly general answers without citing the original reference [22,28,60].

#### Bias

It has been noted that ChatGPT has issues with disparity and bias among different populations. In other words, because certain groups of people have financial, readability, and accessibility barriers using LLMs, their outcomes of using LLMs will be divergent from others. For example, ChatGPT may exert some financial disparity on the users: unlike previous versions such as GPT-3.5, access to GPT-4 involves a monthly fee [53]. These constraints potentially pose financial barriers, limiting widespread adoption and use of the newer, more advanced models in health care applications.

Moreover, the readability of an LLM’s response may further accentuate health disparity [47]. LLMs such as ChatGPT include texts from scientific websites (eg, Wikipedia) as their training data, which makes their responses sound professional and sophisticated. However, LLMs may produce biased results [6,64], making regulations to prevent bias necessary [17,55].

Furthermore, the training data can also be biased. Since recent LLMs are trained based on human-generated texts from the internet, they also tend to provide biased answers [4]. Besides, algorithms may reinforce current health disparities and inequities [67]. Indeed, outputs from ChatGPT have been shown to be biased in terms of gender, race, and religion [4].

#### Privacy

Privacy issues are important when training or using LLMs in health care settings [6,7,64,77]. All AI systems, including LLMs in health settings, should comply with privacy regulations, including compliance with the Health Insurance Portability and Accountability Act, and implement robust safeguards to ensure the protection of sensitive patient information [6,7,64]. Specifically, LLMs have 3 privacy problems. First, the responses from LLMs may embed training examples directly, which breaches privacy if the training examples are identifiable. Second, LLMs may be susceptible to inferential disclosure. For example, a patient’s membership in a dataset or sensitive attributes may be inferred from LLMs’ responses. Third, it may not be clear whether text data are sufficiently deidentified for the anticipated recipients (which may be anyone in the world) when training LLMs. For instance, we may be able to deidentify text in a manner that sufficiently thwarts people who are not incentivized to attack the system, but we may not be addressing recipients who run machine-assisted attacks.

#### Public Acceptance

Public acceptance, the trust of the public in the application of LLMs in health care, has been mentioned in a study by Shahsavar and Choudhary [3]. A cross-sectional, survey-based study shows that 77.9% (371/476) participants claim that they trust ChatGPT’s diagnosis, most of whom possess a bachelor’s or even master’s degree [3]. People are inclined to trust this new technique when using ChatGPT, partially due to the convenience of obtaining information and the patients’ inclination to search for information [3].

## Discussion

### Principal Findings

This systematic review shows that LLMs have been applied to summarization, medical knowledge inquiry, prediction, and administration. At the same time, there are 4 major themes of concern when using these models in practice, including reliability, bias, privacy, and public acceptance. Specifically, the most popular application (30/65, 46% papers) for LLMs was for medical knowledge inquiries, with the second most popular (25/65, 38%) being summarization, followed by prediction (22/65, 34%), and then administration (9/65, 14%). At the same time, 55 (85%) papers expressed concerns about reliability, 16 (25%) about bias, 6 (9%) about privacy, and 4 (6%) about public acceptance.

### Applications

According to our systematic review, LLMs were heavily applied in summarization and medical knowledge inquiry tasks. The former is probably due to the training method of LLMs, which focuses on their capability to summarize documents and paraphrase paragraphs. The latter is due to the inclusion of general medical knowledge in the training data. Specifically, in the category of summarization, summarizing medical notes is the type of task in which LLMs were applied the most. This is probably due to the simplicity of the task and the existence of redundancy in those notes. By contrast, in the genre of medical knowledge inquiry, taking standard medical exams is the type of task in which LLMs were applied the most. This is probably due to the existence of medical questions and answers on the internet that have been included in the training data of some LLMs, such as ChatGPT.

LLMs were applied in prediction tasks as well. Specifically, in the category of prediction, diagnosis is the type of task in which LLMs were applied but with the most reliability concerns. This is probably because diagnosis is a complex process in comparison with summarization and the current popular LLMs (eg, ChatGPT) used insufficient publicly available health datasets for model training. It might also be due to poorly constructed prompts without enough accurate information. Thus, LLMs are still not likely to be suitable for generating reliable answers to uncommon questions. In the category of administration, LLMs were applied equally heavily in various tasks, such as appointment scheduling, information collection, and documentation.

### Concerns

For those applications of LLMs in health care, the 2 greatest concerns are reliability and bias (including disparity and inequality). These concerns might eventually drive this application away from practical implementation.

Notably, about 85% (55/65) of the reviewed studies emphasized concerns about the reliability of LLMs’ responses given that they may impact a patient’s health-related behavior. The concerns about reliability arose mainly from 2 aspects: the quality of the training data in terms of data source and data timeliness, and the models themselves in terms of their performance (eg, accuracy). For example, GPT-3.5 was pretrained using data collected by September 2021, and it also does not have access to private health records. Furthermore, most data that are used to train LLMs are crawled from the internet rather than professionally validated sources. In addition, the generative nature of LLM may result in seeming professional writing but fabricating responses. However, according to Shahsavar and Choudhury [3], people are inclined to trust this new technique, due partially to the convenience of obtaining information and the patients’ inclination to search for information. By contrast, LLMs exhibit mixed predictive performance across different applications. In critical scenarios where incorrect predictions could lead to fatalities, even a 15% difference in accuracy from the gold standard (eg, 59/60, 98% vs 25/30, 83%) [4] could significantly hinder their use in real-world applications.

The issue of bias (or disparity) is mentioned in about 25% (16/65) of our included references. LLM biases come from the training stage (eg, biased training data and biased algorithms) and the application stage (eg, biased user base and biased outcomes). These papers discussed biases mainly from 3 different aspects: financial costs, readability, and accessibility. For example, Hirosawa et al [4] pointed out that the bias encoded in human-generated texts will make LLMs generate biased output; Lee et al [78] concerned that health disparity may result from low readability made by the sophistication of LLM wording; and Johnson et al [48] noted that LLM algorithms tend to reinforce the health disparity and to prevent LLM algorithms from exacerbating current disparity in health.

Another concern that prevents the wide application of LLMs in health care is privacy. When using third-party LLMs, such as ChatGPT, health care organizations face several privacy issues. Although no privacy breach of LLMs regarding patient information has been reported, attacks for other types of private information targeting ChatGPT have been found [79]. For example, a breach led to the exposure of users’ conversations to unauthorized parties [79]. As ChatGPT interacts with patients directly, it may gather personal health information and may breach their privacy [7]. Therefore, many medical centers do not allow researchers and health care providers to use raw patient data as inputs to ChatGPT and other LLMs or even ban their access to these services during work [80]. Training or fine-tuning open-source LLMs requires a large amount of clinical data, which may lead to violations of patients’ privacy, perhaps inadvertently [6,37,64].

### Limitations of the Reviewed Papers

The reviewed papers demonstrated 2 common limitations of their approaches. First, almost all the studies relied on human experts to rate LLMs’ responses. This is problematic because the score may be subjective and more likely unrepresentative. Correspondingly, future works can focus on designing a formal and fair process to evaluate LLMs’ responses from a broad range of stakeholders, including researchers, health care providers, patients, or any users with diverse medical and sociodemographic backgrounds. Second, some of the concerns mentioned in this review (eg, bias) are merely researchers’ speculations of the potential risks that were included to provide directions for further work. However, the mechanisms of how the training of LLMs leads to such concerns have not been comprehensively examined through experiments. It is suggested the audience should be wary of taking these concerns for granted or as proven facts.

### Opportunities

Among all the included papers, few of them propose solutions to improve the reliability of LLMs. First, future research work should focus more on how to improve the accuracy of LLMs’ responses in the health care domain. More specifically, domain-specific health data are demanded for training and fine-tuning of LLMs to improve the performance of LLMs in various tasks in the health care domain. Therefore, data harmonization and consortia established for LLM training are potential directions that can benefit the broad research community. Qualified medical professionals can contribute to the creation of the dataset for LLM training. This, however, will be expensive in terms of time and effort [2]. Alternatively, using retrieval-augmented generation to augment LLM with external knowledge that is up-to-date might be a solution for scenarios where accurate, in-depth professorial knowledge is required. Second, to prevent the hallucination issue, LLMs should be limited to making responses based on validated references. Blockchain technology can be used in this process to provide validation and traceability. Moreover, a holistic system, or a keep-experts-in-the-loop framework that efficiently facilitates the expert validation process becomes important to improve the accuracy and safety of health LLMs. Third, clinical trials based on health outcomes, such as mortality rates, should be conducted to validate the utility of LLM applications formally [1].

How conversational LLMs lead to bias or privacy issues in health care research was not thoughtfully examined with experiments in our reviewed papers. Future studies should first focus on investigating the mechanisms of how LLMs caused bias and privacy issues with stringent experiments and then developing practical solutions.

Regarding bias issues, it is suggested that systematic monitoring is necessary to ensure the impartial functioning of LLMs. However, all these sources discuss bias only with mere sentences and superficial summaries without any experimental investigation. Hence, it is worth noting that further work should also focus more on conducting experiments to understand how bias impacts the responses of LLMs in information, diagnosis, recommendation, and surveillance. More specifically, all applications of LLMs in health care should be tested regarding the exhibitions of bias and the bias mitigation strategies, such as data augmentation and targeted recruitment (eg, the All of Us Research Program targets the collection of data from historically underrepresented populations [81]).

Regarding privacy issues, 2 technical approaches to mitigate the privacy risk while training LLMs are data anonymization [82] and synthetic data generation [83]. For deep learning models, model inversion attacks can potentially infer training data by giving model weights [84]. Considering the exponentially increased open-sourced LLMs with published model weights, a sensitive patient dataset needs to be deidentified [85] or replaced with a synthetic dataset before being used to train or fine-tune an LLM. Otherwise, the patients with whom the data are associated should be informed about their participation in the training or fine-tuning process [86]. To solve the privacy issues, legal, social, and technical protection approaches need to be implemented together to ensure the privacy and security of the whole process of training and using LLMs for health care applications [87].

To raise the public acceptance level of LLMs, explainable AI should be used to address the interpretability issues of LLMs by making the training data and model architecture transparent. More rigorous experimental studies using LLMs are encouraged in the “AI in medicine” research community to demonstrate or improve the reliability of LLM applications in health care. Moreover, stakeholders and decision-makers can propose new policies or regulations to manage the accountability and transparency of AI-generated content, including the responses from LLMs.

There appears to be research that is beginning to address some of these raised issues. For example, Zack et al [88] assessed the potential of GPT-4 to perpetuate racial and gender biases in health care. Hanna et al [89] assessed the racial bias of ChatGPT in health care–related text generation tasks. However, more research studies in these directions are needed to validate these findings and conduct more comprehensive and transparent assessments.

Furthermore, it is important to consider the interconnections among these categories of concerns. For instance, privacy protection methods may negatively affect the quality of training data and, consequently, the model’s reliability due to the tradeoff between data utility and privacy [90-96]. In addition, tackling privacy issues can influence model fairness in different ways, depending on the approach used [93,97,98]. Therefore, we recommend addressing these concerns holistically rather than in isolation.

Moreover, almost all the research studies LLMs’ responses in 1 language. For example, 95% (62/65) of studies focus on English, 1 (2%) focuses on Korean [2], 1 (2%) focuses on Chinese [62], and 1 (2%) focuses on Japanese [50]. Their findings cannot be extrapolated to other languages directly. Considering that many patients or people around the world or even in the United States do not speak English, it is necessary to guarantee that LLMs are usable universally or equitably and conduct more research to investigate the performance of LLMs in other languages.

### Limitations of This Review

Despite notable findings, this review has several limitations. Firstly, the review used PubMed, ACM Digital Library, and IEEE Xplore as the primary sources for the papers. Other sources, such as Scopus, Web of Science, ScienceDirect, or non-English sources, may provide additional candidate papers regarding LLMs for health. However, because PubMed is the main digital library for medical publications, the research findings of this review should be valuable to health care researchers or policy makers. Second, although this review intended to study the application of state-of-the-art conversational LLMs in health care, most of the papers included are about ChatGPT. This is because ChatGPT is still the most powerful conversational LLM. However, its closed-source nature, which is against its company name—OpenAI—may be a hurdle to its wide application in health care, due primarily to the privacy concern when sharing sensitive patient information within prompts with OpenAI. Third, our search terms did not include any medicine-related keywords, which may have limited the number of papers included in this review. Finally, only peer-reviewed papers published before September 2023 are included in our review. Therefore, on one hand, the latest LLM application developments in this area are not included in this review. Specifically, papers focused on LLMs other than ChatGPT, such as LLaMA, were very limited in our initial keyword search results, and only a few of them are included in this review. This is a problem because, while monomodal conversational LLMs have been applied to many fields in health care, the multimodal LLMs that can process medical images, such as GPT-4, Large Language and Vision Assistant (LLaVA) [99] based on LLaMA, and LLaVA-Med [100] based on LLaVA, were just released before September 2023 and are still being examined by researchers regarding their capabilities in health care research. Therefore, no peer-reviewed research papers about applications of multimodal LLMs in health care have been published before September 2023. The main challenge of the application of multimodal LLMs in health care is that multimodal LLMs are still not perfect, either due to insufficient training data or due to insufficient model parameters. Specifically, with the development of computing power, reduced computing cost, and reduced data access cost, LLMs can be applied to multimedia-based diagnosis and analysis in radiology and other departments. By contrast, the latest studies addressing the concerns are not included in this review. Although there is research that is beginning to address some of the issues raised in the systematic review [13,89], there may not have been sufficient time for all recent papers to be deposited into the repositories upon which this investigation relied yet.

### Conclusions

This review summarized applications of the state-of-the-art conversational LLMs in health care and the concerns that need to be resolved in the future. According to the reviewed research articles, conversational LLMs perform well in summarizing health-related texts, answering general questions in health care, and collecting information from patients. However, their performance is relatively less satisfying in making diagnoses and offering recommendations based on patients’ symptoms and other information. Most authors were concerned about the accuracy and consistency of the LLM responses, which should be the primary issues that researchers need to address in the near future. Nevertheless, other concerns regarding bias and privacy issues also prevent conversational LLMs from being broadly applied in the health care domain. However, these concerns still receive insufficient attention: few studies examine the bias and privacy issues in LLMs’ health-related applications with rigorous scientific experiments. Future research should focus more on conducting such research to investigate the mechanisms of how the training and application of conversational LLMs leads to such concerns and to address these concerns that have been seen on any AI tools so that they can be safely applied in the health care domain.

## Appendix

Multimedia Appendix 1 PRISMA (Preferred Reporting Items for Systematic Reviews and Meta-Analyses) checklist.

## Abbreviations

AI: artificial intelligence
BERT: Bidirectional Encoder Representations From Transformers
LLaVA: Large Language and Vision Assistant
LLM: large language model
PRISMA: Preferred Reporting Items for Systematic Reviews and Meta-Analyses

## Data Availability

All data generated or analyzed during this study are included in this published article (Table 1).

## References

[1] Thirunavukarasu AJ, Ting DS, Elangovan K, Gutierrez L, Tan TF, Ting DS (2023). Large language models in medicine. Nat Med.

[2] Jo E, Epstein DA, Jung H, Kim YH (2023). Understanding the benefits and challenges of deploying conversational AI leveraging large language models for public health intervention. Proceedings of the 2023 CHI Conference on Human Factors in Computing Systems.

[3] Shahsavar Y, Choudhury A (2023). User intentions to use ChatGPT for self-diagnosis and health-related purposes: cross-sectional survey study. JMIR Hum Factors.

[4] Hirosawa T, Harada Y, Yokose M, Sakamoto T, Kawamura R, Shimizu T (2023). Diagnostic accuracy of differential-diagnosis lists generated by generative pretrained transformer 3 chatbot for clinical vignettes with common chief complaints: a pilot study. Int J Environ Res Public Health.

[5] Anghelescu A, Firan FC, Onose G, Munteanu C, Trandafir AI, Ciobanu I, Gheorghița Ș, Ciobanu V (2023). PRISMA Systematic literature review, including with meta-analysis vs. chatbot/GPT (AI) regarding current scientific data on the main effects of the calf blood deproteinized hemoderivative medicine (Actovegin) in ischemic stroke. Biomedicines.

[6] Huang H, Zheng O, Wang D, Yin J, Wang Z, Ding S, Yin H, Xu C, Yang R, Zheng Q, Shi B (2023). ChatGPT for shaping the future of dentistry: the potential of multi-modal large language model. Int J Oral Sci.

[7] Wang C, Liu S, Yang H, Guo J, Wu Y, Liu J (2023). Ethical considerations of using ChatGPT in health care. J Med Internet Res.

[8] Sallam M (2023). ChatGPT utility in healthcare education, research, and practice: systematic review on the promising perspectives and valid concerns. Healthcare (Basel).

[9] Park YJ, Pillai A, Deng J, Guo E, Gupta M, Paget M, Naugler C (2024). Assessing the research landscape and clinical utility of large language models: a scoping review. BMC Med Inform Decis Mak.

[10] Giannakopoulos K, Kavadella A, Aaqel Salim A, Stamatopoulos V, Kaklamanos EG (2023). Evaluation of the performance of generative AI large language models ChatGPT, Google Bard, and Microsoft Bing chat in supporting evidence-based dentistry: comparative mixed methods study. J Med Internet Res.

[11] Puladi B, Gsaxner C, Kleesiek J, Hölzle F, Röhrig R, Egger J (2024). The impact and opportunities of large language models like ChatGPT in oral and maxillofacial surgery: a narrative review. Int J Oral Maxillofac Surg.

[12] Pool J, Indulska M, Sadiq S (2024). Large language models and generative AI in telehealth: a responsible use lens. J Am Med Inform Assoc.

[13] Meskó B, Topol EJ (2023). The imperative for regulatory oversight of large language models (or generative AI) in healthcare. NPJ Digit Med.

[14] Sarkis-Onofre R, Catalá-López F, Aromataris E, Lockwood C (2021). How to properly use the PRISMA Statement. Syst Rev.

[15] Moher D, Liberati A, Tetzlaff J, Altman DG, PRISMA Group (2009). Preferred reporting items for systematic reviews and meta-analyses: the PRISMA statement. PLoS Med.

[16] Akhter HM, Cooper JS (2023). Acute pulmonary edema after hyperbaric oxygen treatment: a case report written with ChatGPT assistance. Cureus.

[17] Almazyad M, Aljofan F, Abouammoh NA, Muaygil R, Malki KH, Aljamaan F, Alturki A, Alayed T, Alshehri SS, Alrbiaan A, Alsatrawi M, Temsah HA, Alsohime F, Alhaboob AA, Alabdulhafid M, Jamal A, Alhasan K, Al-Eyadhy A, Temsah MH (2023). Enhancing expert panel discussions in pediatric palliative care: innovative scenario development and summarization with ChatGPT-4. Cureus.

[18] Bhattacharyya M, Miller VM, Bhattacharyya D, Miller LE (2023). High rates of fabricated and inaccurate references in ChatGPT-generated medical content. Cureus.

[19] Bosbach WA, Senge JF, Nemeth B, Omar SH, Mitrakovic M, Beisbart C, Horváth A, Heverhagen J, Daneshvar K (2024). Ability of ChatGPT to generate competent radiology reports for distal radius fracture by use of RSNA template items and integrated AO classifier. Curr Probl Diagn Radiol.

[20] Chen X, Zhang X, Liu Y, Wang Z, Zhou Y, Chu M (2023). RISK-GPT: using ChatGPT to construct a reliable risk factor database for all known diseases. J Glob Health.

[21] Lahat A, Shachar E, Avidan B, Shatz Z, Glicksberg BS, Klang E (2023). Evaluating the use of large language model in identifying top research questions in gastroenterology. Sci Rep.

[22] Puthenpura V, Nadkarni S, DiLuna M, Hieftje K, Marks A (2023). Personality changes and staring spells in a 12-year-old child: a case report incorporating ChatGPT, a natural language processing tool driven by artificial intelligence (AI). Cureus.

[23] Robinson A, Aggarwal S (2023). When precision meets penmanship: ChatGPT and surgery documentation. Cureus.

[24] Zhou Z (2023). Evaluation of ChatGPT's capabilities in medical report generation. Cureus.

[25] Guirguis CA, Crossley JR, Malekzadeh S (2023). Bilateral vocal fold paralysis in a patient with neurosarcoidosis: a ChatGPT-driven case report describing an unusual presentation. Cureus.

[26] Haemmerli J, Sveikata L, Nouri A, May A, Egervari K, Freyschlag C, Lobrinus JA, Migliorini D, Momjian S, Sanda N, Schaller K, Tran S, Yeung J, Bijlenga P (2023). ChatGPT in glioma adjuvant therapy decision making: ready to assume the role of a doctor in the tumour board?. BMJ Health Care Inform.

[27] Lyu Q, Tan J, Zapadka ME, Ponnatapura J, Niu C, Myers KJ, Wang G, Whitlow CT (2023). Translating radiology reports into plain language using ChatGPT and GPT-4 with prompt learning: results, limitations, and potential. Vis Comput Ind Biomed Art.

[28] Cunningham AR, Behm HE, Ju A, Peach MS (2023). Long-term survival of patients with glioblastoma of the pineal gland: a ChatGPT-assisted, updated case of a multimodal treatment strategy resulting in extremely long overall survival at a site with historically poor outcomes. Cureus.

[29] Golan R, Ripps SJ, Reddy R, Loloi J, Bernstein AP, Connelly ZM, Golan NS, Ramasamy R (2023). ChatGPT's ability to assess quality and readability of online medical information: evidence from a cross-sectional study. Cureus.

[30] Readable homepage. Readable.

[31] Hamed E, Sharif A, Eid A, Alfehaidi A, Alberry M (2023). Advancing artificial intelligence for clinical knowledge retrieval: a case study using ChatGPT-4 and link retrieval plug-in to analyze diabetic ketoacidosis guidelines. Cureus.

[32] Grewal H, Dhillon G, Monga V, Sharma P, Buddhavarapu VS, Sidhu G, Kashyap R (2023). Radiology gets chatty: the ChatGPT saga unfolds. Cureus.

[33] Kumari KS, Anusha KS (2023). An esthetic approach for rehabilitation of long-span edentulous arch using artificial intelligence. Cureus.

[34] Cadamuro J, Cabitza F, Debeljak Z, De Bruyne S, Frans GM, Perez SM, Ozdemir H, Tolios A, Carobene A, Padoan A (2023). Potentials and pitfalls of ChatGPT and natural-language artificial intelligence models for the understanding of laboratory medicine test results. An assessment by the European Federation of Clinical Chemistry and Laboratory Medicine (EFLM) Working Group on Artificial Intelligence (WG-AI). Clin Chem Lab Med.

[35] Jiang LY, Liu XC, Nejatian NP, Nasir-Moin M, Wang D, Abidin A, Eaton K, Riina HA, Laufer I, Punjabi P, Miceli M, Kim NC, Orillac C, Schnurman Z, Livia C, Weiss H, Kurland D, Neifert S, Dastagirzada Y, Kondziolka D, Cheung AT, Yang G, Cao M, Flores M, Costa AB, Aphinyanaphongs Y, Cho K, Oermann EK (2023). Health system-scale language models are all-purpose prediction engines. Nature.

[36] Sharma B, Gao Y, Miller T, Churpek M, Afshar M, Dligach D (2023). Multi-task training with in-domain language models for diagnostic reasoning. Proceedings of the 5th Clinical Natural Language Processing Workshop.

[37] Liu J, Wang C, Liu S (2023). Utility of ChatGPT in clinical practice. J Med Internet Res.

[38] Hamed E, Eid A, Alberry M (2023). Exploring ChatGPT's potential in facilitating adaptation of clinical guidelines: a case study of diabetic ketoacidosis guidelines. Cureus.

[39] Kim HY (2023). A case report on ground-level alternobaric vertigo due to eustachian tube dysfunction with the assistance of conversational generative pre-trained transformer (ChatGPT). Cureus.

[40] Macdonald C, Adeloye D, Sheikh A, Rudan I (2023). Can ChatGPT draft a research article? An example of population-level vaccine effectiveness analysis. J Glob Health.

[41] Cascella M, Montomoli J, Bellini V, Bignami E (2023). Evaluating the feasibility of ChatGPT in healthcare: an analysis of multiple clinical and research scenarios. J Med Syst.

[42] Ali MJ (2023). ChatGPT and lacrimal drainage disorders: performance and scope of improvement. Ophthalmic Plast Reconstr Surg.

[43] Antaki F, Touma S, Milad D, El-Khoury J, Duval R (2023). Evaluating the performance of ChatGPT in ophthalmology: an analysis of its successes and shortcomings. Ophthalmol Sci.

[44] Bird JJ, Lotfi A (2023). Generative transformer chatbots for mental health support: a study on depression and anxiety. Proceedings of the 16th International Conference on PErvasive Technologies Related to Assistive Environments.

[45] Hoch CC, Wollenberg B, Lüers JC, Knoedler S, Knoedler L, Frank K, Cotofana S, Alfertshofer M (2023). ChatGPT's quiz skills in different otolaryngology subspecialties: an analysis of 2576 single-choice and multiple-choice board certification preparation questions. Eur Arch Otorhinolaryngol.

[46] Holmes J, Liu Z, Zhang L, Ding Y, Sio TT, McGee LA, Ashman JB, Li X, Liu T, Shen J, Liu W (2023). Evaluating large language models on a highly-specialized topic, radiation oncology physics. Front Oncol.

[47] Hristidis V, Ruggiano N, Brown EL, Ganta SR, Stewart S (2023). ChatGPT vs Google for queries related to dementia and other cognitive decline: comparison of results. J Med Internet Res.

[48] Johnson SB, King AJ, Warner EL, Aneja S, Kann BH, Bylund CL (2023). Using ChatGPT to evaluate cancer myths and misconceptions: artificial intelligence and cancer information. JNCI Cancer Spectr.

[49] Kung TH, Cheatham M, Medenilla A, Sillos C, De Leon L, Elepaño C, Madriaga M, Aggabao R, Diaz-Candido G, Maningo J, Tseng V (2023). Performance of ChatGPT on USMLE: potential for AI-assisted medical education using large language models. PLOS Digit Health.

[50] Kusunose K, Kashima S, Sata M (2023). Evaluation of the accuracy of ChatGPT in answering clinical questions on the Japanese Society of Hypertension Guidelines. Circ J.

[51] Lahat A, Shachar E, Avidan B, Glicksberg B, Klang E (2023). Evaluating the utility of a large language model in answering common patients' gastrointestinal health-related questions: are we there yet?. Diagnostics (Basel).

[52] Li Y, Li Z, Zhang K, Dan R, Jiang S, Zhang Y (2023). ChatDoctor: a medical chat model fine-tuned on a Large Language Model Meta-AI (LLaMA) using medical domain knowledge. Cureus.

[53] Moshirfar M, Altaf AW, Stoakes IM, Tuttle JJ, Hoopes PC (2023). Artificial intelligence in ophthalmology: a comparative analysis of GPT-3.5, GPT-4, and human expertise in answering StatPearls questions. Cureus.

[54] Nov O, Singh N, Mann D (2023). Putting ChatGPT's medical advice to the (Turing) test: survey study. JMIR Med Educ.

[55] Sallam M, Salim NA, Al-Tammemi AB, Barakat M, Fayyad D, Hallit S, Harapan H, Hallit R, Mahafzah A (2023). ChatGPT output regarding compulsory vaccination and COVID-19 vaccine conspiracy: a descriptive study at the outset of a paradigm shift in online search for information. Cureus.

[56] Sinha RK, Deb Roy A, Kumar N, Mondal H (2023). Applicability of ChatGPT in assisting to solve higher order problems in pathology. Cureus.

[57] Thirunavukarasu AJ, Hassan R, Mahmood S, Sanghera R, Barzangi K, El Mukashfi M, Shah S (2023). Trialling a large language model (ChatGPT) in general practice with the applied knowledge test: observational study demonstrating opportunities and limitations in primary care. JMIR Med Educ.

[58] Van Bulck L, Moons P (2024). What if your patient switches from Dr. Google to Dr. ChatGPT? A vignette-based survey of the trustworthiness, value, and danger of ChatGPT-generated responses to health questions. Eur J Cardiovasc Nurs.

[59] Wagner MW, Ertl-Wagner BB (2024). Accuracy of information and references using ChatGPT-3 for retrieval of clinical radiological information. Can Assoc Radiol J.

[60] Walker HL, Ghani S, Kuemmerli C, Nebiker CA, Müller BP, Raptis DA, Staubli SM (2023). Reliability of medical information provided by ChatGPT: assessment against clinical guidelines and patient information quality instrument. J Med Internet Res.

[61] Yeo YH, Samaan JS, Ng WH, Ting PS, Trivedi H, Vipani A, Ayoub W, Yang JD, Liran O, Spiegel B, Kuo A (2023). Assessing the performance of ChatGPT in answering questions regarding cirrhosis and hepatocellular carcinoma. Clin Mol Hepatol.

[62] Zhu Z, Ying Y, Zhu J, Wu H (2023). ChatGPT's potential role in non-English-speaking outpatient clinic settings. Digit Health.

[63] Altamimi I, Altamimi A, Alhumimidi AS, Altamimi A, Temsah MH (2023). Snakebite advice and counseling from artificial intelligence: an acute venomous snakebite consultation with ChatGPT. Cureus.

[64] Goktas P, Karakaya G, Kalyoncu AF, Damadoglu E (2023). Artificial intelligence chatbots in allergy and immunology practice: where have we been and where are we going?. J Allergy Clin Immunol Pract.

[65] Chervenak J, Lieman H, Blanco-Breindel M, Jindal S (2023). The promise and peril of using a large language model to obtain clinical information: ChatGPT performs strongly as a fertility counseling tool with limitations. Fertil Steril.

[66] Agbavor F, Liang H (2022). Predicting dementia from spontaneous speech using large language models. PLOS Digit Health.

[67] Karkera N, Acharya S, Palaniappan SK (2023). Leveraging pre-trained language models for mining microbiome-disease relationships. BMC Bioinformatics.

[68] Sarbay İ, Berikol GB, Özturan IU (2023). Performance of emergency triage prediction of an open access natural language processing based chatbot application (ChatGPT): a preliminary, scenario-based cross-sectional study. Turk J Emerg Med.

[69] Galido PV, Butala S, Chakerian M, Agustines D (2023). A case study demonstrating applications of ChatGPT in the clinical management of treatment-resistant schizophrenia. Cureus.

[70] Sorin V, Klang E, Sklair-Levy M, Cohen I, Zippel DB, Balint Lahat N, Konen E, Barash Y (2023). Large language model (ChatGPT) as a support tool for breast tumor board. NPJ Breast Cancer.

[71] Juhi A, Pipil N, Santra S, Mondal S, Behera JK, Mondal H (2023). The capability of ChatGPT in predicting and explaining common drug-drug interactions. Cureus.

[72] Haver HL, Ambinder EB, Bahl M, Oluyemi ET, Jeudy J, Yi PH (2023). Appropriateness of breast cancer prevention and screening recommendations provided by ChatGPT. Radiology.

[73] Liu S, Wright AP, Patterson BL, Wanderer JP, Turer RW, Nelson SD, McCoy AB, Sittig DF, Wright A (2023). Using AI-generated suggestions from ChatGPT to optimize clinical decision support. J Am Med Inform Assoc.

[74] Kao HJ, Chien TW, Wang WC, Chou W, Chow JC (2023). Assessing ChatGPT's capacity for clinical decision support in pediatrics: a comparative study with pediatricians using KIDMAP of Rasch analysis. Medicine (Baltimore).

[75] Schulte B (2023). Capacity of ChatGPT to identify guideline-based treatments for advanced solid tumors. Cureus.

[76] Carpenter KA, Altman RB (2023). Using GPT-3 to build a lexicon of drugs of abuse synonyms for social media pharmacovigilance. Biomolecules.

[77] Montagna S, Ferretti S, Klopfenstein LC, Florio A, Pengo M (2023). Data decentralisation of LLM-based chatbot systems in chronic disease self-management. Proceedings of the 2023 ACM Conference on Information Technology for Social Good.

[78] Lee TC, Staller K, Botoman V, Pathipati MP, Varma S, Kuo B (2023). ChatGPT answers common patient questions about colonoscopy. Gastroenterology.

[79] Gupta M, Akiri C, Aryal K, Parker E, Praharaj L (2023). From ChatGPT to ThreatGPT: impact of generative AI in cybersecurity and privacy. IEEE Access.

[80] Nelson F (2023). Many companies are banning ChatGPT. This is why. Science Alert.

[81] The All of Us Research Program Investigators (2019). The “All of Us” research program. N Engl J Med.

[82] El Emam K, Rodgers S, Malin B (2015). Anonymising and sharing individual patient data. BMJ.

[83] Chen RJ, Lu MY, Chen TY, Williamson DF, Mahmood F (2021). Synthetic data in machine learning for medicine and healthcare. Nat Biomed Eng.

[84] Zhang Y, Jia R, Pei H, Wang W, Li B, Song D (2020). The secret revealer: generative model-inversion attacks against deep neural networks. Proceedings of the IEEE/CVF Conference on Computer Vision and Pattern Recognition.

[85] El Emam K, Jonker E, Arbuckle L, Malin B (2011). A systematic review of re-identification attacks on health data. PLoS One.

[86] Cohen IG (2023). What should ChatGPT mean for bioethics?. Am J Bioeth.

[87] Meskó Bertalan, Topol EJ (2023). The imperative for regulatory oversight of large language models (or generative AI) in healthcare. NPJ Digit Med.

[88] Zack T, Lehman E, Suzgun M, Rodriguez JA, Celi LA, Gichoya J, Jurafsky D, Szolovits P, Bates DW, Abdulnour RE, Butte AJ, Alsentzer E (2024). Assessing the potential of GPT-4 to perpetuate racial and gender biases in health care: a model evaluation study. Lancet Digit Health.

[89] Hanna JJ, Wakene AD, Lehmann CU, Medford RJ Assessing racial and ethnic bias in text generation for healthcare-related tasks by ChatGPT. medRxiv.

[90] Wan Z, Vorobeychik Y, Xia W, Clayton EW, Kantarcioglu M, Ganta R, Heatherly R, Malin BA (2015). A game theoretic framework for analyzing re-identification risk. PLoS One.

[91] Wan Z, Vorobeychik Y, Xia W, Clayton EW, Kantarcioglu M, Malin B (2017). Expanding access to large-scale genomic data while promoting privacy: a game theoretic approach. Am J Hum Genet.

[92] Wan Z, Vorobeychik Y, Kantarcioglu M, Malin B (2017). Controlling the signal: practical privacy protection of genomic data sharing through Beacon services. BMC Med Genomics.

[93] Wan Z, Vorobeychik Y, Xia W, Liu Y, Wooders M, Guo J, Yin Z, Clayton EW, Kantarcioglu M, Malin BA (2021). Using game theory to thwart multistage privacy intrusions when sharing data. Sci Adv.

[94] Wan Z, Hazel JW, Clayton EW, Vorobeychik Y, Kantarcioglu M, Malin BA (2022). Sociotechnical safeguards for genomic data privacy. Nat Rev Genet.

[95] Yan C, Yan Y, Wan Z, Zhang Z, Omberg L, Guinney J, Mooney SD, Malin BA (2022). A multifaceted benchmarking of synthetic electronic health record generation models. Nat Commun.

[96] Venkatesaramani R, Wan Z, Malin BA, Vorobeychik Y (2023). Enabling tradeoffs in privacy and utility in genomic data Beacons and summary statistics. Genome Res.

[97] Brown JT, Clayton EW, Matheny M, Kantarcioglu M, Vorobeychik Y, Malin BA (2024). Robin Hood: a de-identification method to preserve minority representation for disparities research. Proceedings of the International Conference on Privacy in Statistical Databases.

[98] Zhang T, Zhu T, Gao K, Zhou W, Yu PS (2023). Balancing learning model privacy, fairness, and accuracy with early stopping criteria. IEEE Trans Neural Netw Learn Syst.

[99] Liu H, Li C, Wu Q, Lee YJ Visual instruction tuning. arXiv.

[100] Li C, Wong C, Zhang S, Usuyama N, Liu H, Yang J, Naumann T, Poon H, Gao J LLaVA-Med: training a large language-and-vision assistant for biomedicine in one day. arXiv.

